# Supplementary material for: Enhancing oscillations in intracranial electrophysiological recordings with data-driven spatial filters
Source: PLoS Comput Biol. 2021 Aug 19;17(8):e1009298. doi: 10.1371/journal.pcbi.1009298 (PMC8407590; doi:10.1371/journal.pcbi.1009298)
Supplement: Attachment — Submitted filename: response_to_reviewers.pdf [file pcbi.1009298.s004.pdf]

Response to reviewers

We reply to the comments point-by-point below, with changes to the manuscript given below in blue (additions)/~~red~~ (deletions). Line numbers refer to the manuscript version with highlighted changes.

Reviewer #1:

> This study makes a great case for using spatio-spectral decomposition as a data-driven referencing technique for iEEG as opposed to using methods such as bipolar or Laplacian or common-average referencing. The data-driven nature of this strategy is clearly to its benefit and can be incredibly relevant when signals tend to be spread simultaneously across multiple electrodes, for example, due to volume conduction. While I have been convinced of the value of this method and its usefulness for application to iEEG data, I found aspects of the narrative difficult to follow and several conclusions to be too broad.

We thank the reviewer for taking the time to evaluate our manuscript thoroughly. Their comments helped us to clarify the data generating model, the calculation of spatial patterns and bias and comparisons to other methods in our manuscript.

R1 comment #1

> In the Introduction, the paragraph beginning at line 29 appeared to jump between spatial filtering and source reconstruction. I think reorganizing it in a way that highlights how source reconstruction is also a form of spatial filtering will help make it clearer (maybe):
>
> a. source reconstruction can be reframed as a spatial filtering problem that depends upon several assumptions
> b. past work has built numerous methods that fit into this mold (biophysical modeling, ICA, etc.)
> c. referencing is a particular instance of spatial filtering, and the common referencing strategies used, while appearing to have no assumptions makes strong assumptions about the nature of the sources measured

We thank the reviewer for this suggestion, which helped us clarify the language in the introduction. We have reorganized the text in order to reflect the relationships between spatial filtering, referencing and source reconstruction, see page 2, line 23:

Because the coexistence of different types of neural activity leads to superposition on the signal recorded with electrodes, many different methodological approaches exist to untangle distinct activity sources from electrode signals. One can leverage the multivariate structure of iEEG recordings, in which a number of electrodes are placed on the cortical surface to acquire time series data, toward this end. Each electrode picks up a mixture of signals from different types of cortical sources, determined by location and orientation of the generating sources and the biophysical properties of the tissue. Fig. 1 illustrates the underlying data model for iEEG. This spatial mixing is given by the forward model and is assumed to be linear here (Parra et al., 2005).

For noninvasive electrophysiological recording techniques such as electroencephalography (EEG) and magnetoencephalography (MEG), source reconstruction techniques are commonly used to extract independent activity sources from sensor space data (Jas et al., 2018). Many approaches can be framed as the estimation of spatial filters that satisfy pre-defined optimization criteria, taking into account either biophysical constraints given by cortex morphology or statistical properties of the signals, which for instance are considered when computing principal or independent component analysis. A spatial filter allows computation of a new, filtered signal trace using a weighted sum of all other electrodes. For each time point, the dot product of the electrode data with the spatial filter is taken to yield the corresponding entry for the source trace. The spatial filter vector is the same for all time points and this operation can be performed efficiently by matrix multiplication.

For iEEG recordings, source reconstruction has mostly been employed in the context of localizing epileptic seizure focus, both with biophysical modeling (Pascarella et al.,2016; Dümpelmann et al., 2012; Fuchs et al., 2007, Chintaluri and Wojcik, 2015) and approaches using independent component analysis (Hindriks et al., 2018; Fahimi Hnazaee et al., 2020; Dümpelmann et al., 2012; Hu et al., 2007; Whitmer et al., 2010). But in contrast to non-invasive electrophysiological methods, data referencing techniques dominate for iEEG.

Data referencing can be viewed as the application of a particularly simple spatial filter. For instance, in the case of a bipolar filter, the spatial filter is a vector with as many entries as electrodes, containing weights -1 and +1 for two selected electrodes and zero for all other electrodes. The two most prevalent methods for referencing iEEG data are to apply either a common average reference, with the aim to minimize common noise or distal activity, or to use bipolar reference, with the aim to extract locally generated signals. ~~Source reconstruction for iEEG recordings has mostly employed in the context of localizing epileptic seizure focus. The main techniques used here are biophysical modeling (Pascarella et al., 2016; Dümpelmann et al., 2012;~~

63 Fuchs et al.,2007; Chintaluri and Wojcik, 2015) and approaches using independent component analysis (Hindriks et al., 2018; Michelmann et al., 2018; Fahimi Hnazaee et al., 2020; Dümpelmann et al.,2012; Hu et al.,2007; Whitmer et al., 2010). While a referencing approach is computationally simpler than an approach involving biophysically or statistically constrained source reconstruction, the referencing choice will highly impact the dynamics present in the resulting signal (Liu et al., 2015; Li et al., 2018; Arnulfo et al., 2015; Shirhatti et al., 2016).

For examining high-frequency activity, an electrode-based approach (using a standard common average or bipolar reference) seems to be justified because of limited spatial spread of high-frequency signal content not exceeding inter-electrode distance (Dubey and Ray, 2019; Crone et al., 1998b), with sub-centimeter functional specificity (Flinker et al., 2011). In contrast to that, activity in lower frequency ranges displays an increased spatial spread, showing a high degree of correlation between neighboring electrode locations depending on oscillation frequency (Muller et al., 2016; Crone et al., 1998a). Because of the spatial spread, it is expected that different rhythms contribute to activity of several electrodes due to spatial superposition. Therefore, multivariate separation techniques may improve measurement of cortical rhythms also in iEEG, as for instance was examined using independent component analysis in Michelmann et al. (2018).

Here, we explore a data-driven spatial filtering method, spatio-spectral decomposition (SSD) for specifically extracting oscillatory sources in iEEG data. This technique, based on generalized eigenvalue decomposition, has been shown to be superior to for instance independent component analysis in EEG for extraction of oscillatory sources (Nikulin et al., 2011). Fig. 1 shows the underlying linear model of iEEG data and illustrates bipolar and common average referencing.
For recording iEEG data, a number of electrodes are placed on the cortical surface to acquire time series data. Each electrode picks up a mixture of signals from different types of cortical sources, determined by location and orientation of the generating sources and the biophysical properties of the tissue. This spatial mixing is given by the forward model and is assumed to be linear here (Parra et al., 2005).
The SSD approach helps in recovering distinct estimates distinct putative neuronal sources from the summation activity recorded via the electrodes, i.e., it estimates a backward model in the form of spatial filters with the optimization constraint focussed on a specific frequency band of interest, in order to best measure the temporal dynamics of oscillations in that band and their associated features of interest. Data referencing can be viewed as the application of a spatial filter, in which the trace of each electrode is multiplied with a specific weight. For instance, in the case of a bipolar filter, the spatial filter is a vector with as many entries as electrodes, containing weights -1 and +1 for two selected electrodes and zero for all other electrodes. For each time

~~point, the dot product of the electrode data with the spatial filter is taken to yield the corresponding entry for the component trace. The spatial filter vector is the same for all time points and this operation can be performed efficiently by matrix multiplication.~~ The focus of this approach here is primarily on estimating the source time series. The estimated source time series are subsequently referred to as components. Information about the location of a source is only indirectly provided through the computation of spatial patterns.

R1 comment #2

Referencing has a clear physical basis: that we are estimating voltage gradients. Note also in source reconstruction we are identifying dipole sources, so that the 'filter' applied there leads to clear physical intuition about the outcome. As such, I felt confusion about the physical intuition offered by application of SSD due to the confounding of (i) spatial mixing of multiple independent sources due to volume conduction with (ii) multiple, independent sources all coordinated at/near zero phase. I don't think the two cases can be separated under SSD unless the sources have unique waveforms/unique sources of noise or the results under a Laplacian/Bipolar referencing scheme are compared to SSD. If there is no consistent rhythmic activity in the Laplacian/bipolar but we see weights across multiple electrodes in the SSD this is evidence suggesting that there is a common source.

(a) Lines 46-47: While the idea of spatial superposition of multiple rhythms is relevant to when multiple independent (potentially coherent) rhythms contribute to multiple electrodes, spatial superposition needs to be differentiated from the idea that any single low frequency band contributes to multiple electrodes due to volume conduction (which could equally well happen at low and high frequency bands). The high correlation of low frequency bands may well be due to independent sources becoming coherent rather than a single source that shows up at multiple electrodes. This interpretation is present in lines 77-79, While the first part of the line speaks to rhythms becoming coherent over some spatial area, the second part ('spatial mixing') seems to be speaking to when volume conduction plays a part in what electrodes record, but it is spoken of as though they are the same phenomenon.

In general, the physical intuition regarding the data generating model is the same as for independent component analysis, that the data X is generated by a linear combination of sources S which map onto the electrodes with different loadings: X = AS, as illustrated in Fig. 1. The lines referenced by the reviewer reflect predominantly the superposition of different

rhythms (“spatial mixing of rhythms”) for us, which also involves volume conduction (“activity spread of individual rhythms”).

To clarify our manuscript in that respect, we extended this paragraph to distinguish between those scenarios more clearly in page 5, line 121:

> In this article, we use spatial filters to investigate rhythms present in mainly the alpha and beta-frequency bands in human iEEG recordings. We illustrate two aspects of measuring oscillations in iEEG data. First, that the activity spread of individual rhythms can exceed inter-electrode distance, with single rhythms contributing to several electrodes. Second, ~~and show~~ that spatial mixing of rhythms in intracranial recordings can affect the oscillatory power of a given rhythm as detected on the electrodes and alter its non-sinusoidal waveform shape ~~when sources are mixed~~.

> (b) Lines 58-59: I think the expectation that SSD extracts ‘distinct neural sources’ is not borne out by what the method sets out to do. We simply cannot distinguish between the cases described in (1a) above, so we may have distinct neural sources, but we may not. SSD does appear to summarize the possible sources into a small number.

As SSD works solely using statistical properties, the reviewer is right that two different sources that have phase lag of zero in the majority of time, the covariance matrix will not be decomposable to separate these two rhythms, as mentioned in the limitations section of the discussion. We checked for occurrences of the word ‘recover’ and rephrased in page 3, line 84 (copied from above reply to comment #1) and also see below reply to R1 comment #4:

> The SSD approach ~~helps in recovering~~ estimates distinct neuronal sources from the summation activity recorded via the electrodes, i.e., it estimates a backward model in the form of spatial filters with the optimization constraint focussed on a specific frequency band of interest, in order to best measure the temporal dynamics of oscillations in that band and their associated features of interest.

The reviewer is right that SSD yields a small number of sources, and we have a comment regarding that in the discussion (line 523), looking forward to comparison to higher density electrode setups in the future. It remains to be seen what the limits of resolution are. We have performed some explorations in mouse LFP data, which hint at the fact that source separation techniques will need to incorporate traveling wave like phenomena, to which we have referred to in the limitation section (page 26, line 860), so we hope to contribute future work in this direction.

(c) As such, I think Figure 1 should suggest this more clearly that the sources extracted under SSD are representative, or perhaps equivalent, rather than genuine sources (see Nunez, Nunez, and Srinivasan, 2019, Brain Topogaphy). Line 78-79 also falls prey to this given that SSD can't distinguish between the case of multiple coherent independent sources and the same source spreading to multiple electrodes so really the presence of non-zero weights across multiple electrodes only reveals a consistent rhythm measured across all of them without it necessarily being the same rhythm.

In our view, all sources in EEG/ECoG are equivalent sources, in the terminology of the linked reference. We reflected on our current presentation of Fig. 1, where on the left 'ground truth' sources are depicted and the signals obtained with SSD are labeled 'estimated sources'. We feel that this is an accurate reflection of our conceptual understanding, which stays agnostic to the nature of the sources. We welcome any suggestions on how to modify this figure if the reviewer does not deem this sufficient. We now cite the Nunez et al. reference for a reflection on the word 'source' in the limitation section of the discussion, see page 25, line 808.

Specific limitations of an approach for estimating spatial filters utilizing eigenvalue decomposition are detailed below. First, there is no automatic one-to-one mapping from estimated components onto physiological entities (but neither can this be done from electrode-based activity). In terms of clarifying what these components represent, Nunez et al. (2019) have proposed a distinction between *genuine*, *equivalent* and *representative* sources. Within this framework, the components returned by SSD can be seen as representative sources, not directly reflecting e.g. synaptic activity as in the case of genuine sources, but rather presenting one possibility of many, similar to source estimates returned by independent component analysis. In the case of distinct, but highly co-fluctuating neuronal sources, they will not necessarily be separable on the basis of their covariance. An indication of this are spatial patterns that deviate from the spatial pattern expected for a dipolar source, e.g., by showing several spatially distributed maxima.

R1 comment #3

There are multiple times that source reconstruction is referenced however, this is confusing as it sets up two different goal posts that are alternatively targeted (data-driven referencing vs. agnostically identifying a putative set of representative neural sources). Further, if the goal is to have an approach that approximates a small number of sources from the activity at the electrodes, then multiple alternative approaches should also be demonstrated and compared here akin to Cohen (2017). Additionally, given that source reconstruction involves determining location information

> while incorporating tissue conductivity details, the suggestion that this method identifies sources akin to source reconstruction (as suggested in Figure 1) is a misnomer. I think adding in the caveat that they are representative sources potentially might help with this as well.

As we understand this reviewer comment, it relates to three different aspects:

1) Data-driven referencing & agnostic identification of sources as 2 different goal posts: In our understanding, data-driven referencing and agnostically identifying neural sources are intrinsically linked and cannot be viewed as separate goal posts. Typically, the choice of reference is highly influenced by the type of activity one aims to extract (e.g. focus on local activity for bipolar referencing, or highlighting radial sources for common average referencing). In that sense, our approach aims to amplify a specific type of activity (oscillatory) without preselection of spatial search radius. We searched for the occurrence of the term 'source reconstruction' in the manuscript and it appears in the introduction contrasting anatomically and statistical constrained source analysis approaches for M/EEG and iEEG, so we think the term is sufficiently clear in this context. Additionally, we checked for each occurrence of the term 'source' and substituted 'source time series' or 'source estimates' where this seemed to be more appropriate, and included a note about what we mean with the word component, see the paragraph in point 2) of the reply to this comment. We also hope that the restructuring of the introduction improved the clarity regarding referencing and source reconstruction (see R1 comment #1).

---

2) Misnomer "source reconstruction": We disagree that it is a misnomer to label statistical methods as source reconstruction. In our understanding, source reconstruction is a term that is mainly related to retrieving source *time series*, which is definitely the goal of SSD. We changed the label Figure 1 'estimated sources' to 'estimated source time series', which we feel captures this goal in retrieving the time series. Whereas source localization aims to pinpoint the exact spatial location of the respective source, which is an aspect that is not at the center stage here, and achieved indirectly via the spatial patterns. For EEG, a dipole fit often performed on the spatial patterns to achieve this (Haufe et al. 2014, On the interpretation of weight vectors of linear models in multivariate neuroimaging, Neuroimage), which we did not perform here, as the current dipole approximation is not valid in the light of the ECoG electrode proximity to the tissue. We aim to clarify the distinctions between those two different goals relating to source reconstruction and localization by extending the paragraph in page 3, line 84:

> The SSD approach ~~helps in recovering distinct~~ estimates distinct putative neuronal sources from the summation activity recorded via the electrodes, i.e., it estimates a backward model in the form of spatial filters with the optimization constraint focussed on a specific frequency band of interest, in order to best measure the temporal

dynamics of oscillations in that band and their associated features of interest. The focus of this approach here is primarily on estimating the source time series. The estimated source time series are subsequently referred to as components. Information about the location of a source is only indirectly provided through the computation of spatial patterns.

---

3) Comparing SSD to other similar methods: Regarding comparison to other methods, we think that a simulation based comparison of different methods is outside of the scope of the article, since our goal is not to specifically argue that a certain method is better than another, rather it is focused on introducing this particular class of methods to the iEEG community. Conceptually, SSD is similar to the GEDb method in the Cohen (2017) paper. These two methods are very similar, and we do not expect them to yield different results for the mostly qualitative investigations we performed. Please note that in Cohen (2017), SSD was compared to the other approaches in a peculiar way, by only using the band-pass filtered activity in the respective narrow-band (e.g. alpha) after estimation of spatial filters. In that way all non-sinusoidal properties of the rhythms are lost. This is not how we use SSD in this manuscript here, and SSD will compare very similarly to those approaches, when the spatial filters are applied on broadband data.

We extended the paragraph referencing other methods (page 22, line 681):

> While we chose SSD as a spatial filtering technique for our illustrations, other types of generalized eigenvalue decomposition algorithms are available to solve specific objectives. For enhancing specifically oscillatory SNR, there are variants that maximize the spectral power in a frequency band of interest, compared to the total spectral power (Cheveigné and Arzounian, 2015), ~~which are benchmarked for EEG in (Cohen, 2017),~~ with demonstrations for MEG/EEG as well as monkey ECoG and optical imaging given in (Cheveigné and Parra, 2014) and which are benchmarked for EEG in (Cohen, 2017). Note that in the latter, the benchmark test for SSD only used the band-pass filtered activity for evaluation after application of spatial filters; in this way, many of the non-sinusoidal properties are lost. In our study, we compare the output of SSD using broadband filtered data to preserve nonsinusoidal waveform shape, and we expect other generalized eigenvalue decomposition methods aimed at amplifying oscillatory SNR to perform similarly to SSD when evaluated on broadband (versus narrowband filtered) data. The main aspect we want to highlight here is that generalized eigenvalue decomposition methods are highly flexible and permit interesting contrasts for maximizing/minimizing SNR along specific dimensions. For instance, in the case of task-based data, Common Spatial Patterns (Koles, 1991) maximizes differences between conditions, for instance to investigate main contributions to task-related modulation. [...]

R1 comment #4

In several places I note that there is a desire to draw conclusions about both the data and the benefits of the algorithm at the same time. This, to me, is a difficult inference to perform since ground truth in data is unknown.

In Section 3.1 the conclusion is drawn that since different components from SSD have different spatial patterns (different weights at the same electrode) and slightly different spectra (primarily differing in the 1/f), there are different rhythms at the same electrode. Does past simulation work back this up? Can SSD components be interpreted in this way? I am unclear why this is necessarily true, and why it could be the same rhythm with slightly different noise profiles say. Perhaps a deeper discussion of the implications of the generalized eigenvalue decomposition and what components mean under it (in Section 2.2.2) would clarify this immediately.

This question relates to the underlying data generating model. In general, the simulation work performed, for instance in the original SSD paper (Nikulin et al., 2011) places different dipoles and checks under what circumstances they can be recovered with SSD (result: better than with ICA type methods). To our knowledge, such a linear data generation model is the dominant assumption for electrophysiological data and in the light of such a linear data generating model, we would definitely say that SSD components can be interpreted as arising from separate sources under this data generating model. As pointed out in the limitations section, this data generating model may not be sufficient to capture certain phenomena, like traveling waves.

To follow up on the comment we extended Section 2.2.2 as recommended, regarding the interpretation and properties of the obtained components, see page 8, line 277. (Note: this is the same paragraph that has been updated regarding the related R2 comment #2, please also see this comment, modified paragraph pasted here for convenience).

The number of components returned by SSD is equal to the number of electrodes, with the components ordered by relative SNR in the frequency band of interest. In contrast to PCA, the first few SSD components only capture a small fraction of global variance, as the method is focused on maximizing variance in a specific frequency band. PCA has strong constraints, and can only return a spatial filter matrix W which is orthogonal, i.e., for which $W^T W = Id$ needs to be satisfied. Therefore $W^T = W^{-1} = A$, which means that spatial patterns A are equal to spatial filters for PCA. As this results in spatial filters with high degree of smoothness between neighboring values, PCA will not be able to distinguish rhythms in the same subspace. SSD and other generalized eigenvalue

decomposition methods do not have this constraint. There, the following constraint needs to be satisfied $A^T W = Id$, so the spatial patterns are not generally equal to the spatial filters, as the inverse of a matrix is not generally equal to its transpose. This allows distinguishing sources in the same subspace, e.g. rhythms coming from the same cortical area in the same frequency band. In terms of amplitude of the individual components, they are independent of each other for PCA as well as SSD.

Lines 296-297 suggests that SSD better uses information from the electrodes than other reference strategies. Yes, this is true as SSD is data-driven, but how do the results from the data provide evidence towards this?

We tried to be careful, and generally not argue in terms of ‘better’, but in terms of ‘more flexible’. For instance, our sEEG example in Fig. 3 shows that different fixed referencing will emphasize different aspects of the data, potentially losing information about rhythms of interest. If there is a clearly defined frequency of interest, SSD or other data-driven methods can be of aid here, providing a searchlight to scan for these rhythms specifically.

In line 312, the conclusion that SSD ‘enables recovering of rhythms in a flexible way regardless of location and dipole orientation’ seems an overextended interpretation of the results, this seems to be a conclusion we could have only in the presence of a ground truth (say in a simulation) or a very strong prior. Clearly SSD is agnostic to the biophysical details involved but I’m unsure whether the data can bear this out.

This conclusion is informed by previous studies as outlined in the response to this comment above, but to be more cautious here, we adjusted the text in the paragraph (page 12, line 450)

Additionally, all signals were submitted simultaneously to the SSD procedure, without subselection, making it possible to combine information from multiple leads and electrode configurations efficiently. ~~Using SSD results in less bias due to fixed reference choice, as learning the filter coefficients from the data enables recovery of rhythms in a flexible way regardless of their location and dipole orientation.~~ While standard referencing techniques tend to enhance signals generated via a specific way, SSD is agnostic to the biophysical generation, and can be used more flexibly in this regard. For instance, monopolar and common-average referencing preserve correlation across channels to a higher degree than bipolar referencing (Li et al., 2018). This will influence measurement of rhythms, which can have a different spatial spread across subjects, depending on local cortex anatomy. So, while common average referencing highlights radial sources, bipolar referencing has a focus on locally generated activity and emphasizes bipolar sources. SSD will extract components with maximum SNR agnostic

about their spatial spread. In the next section, we further examine the spatial spread present empirically in iEEG data.

Lines 338-340, this is perhaps something that would be best justified through a simulation/this is a line that might be better in the Discussion with an additional line pointing out that research to define the boundaries of what is possible to be discovered with this method in iEEG through simulations is critical for interpretation of its application.

We agree with the reviewer that our current approach does not allow us to draw this conclusion in the results of our work. Therefore, we have modified the text below as follows, moving the phrase from the results to the discussion, and expanding more on the argument in the discussion to note how future simulation work might help address this.

We have now modified the paragraph to remove that mentioned line (page 15, line 492):

However, in the case of a rhythm of a large spatial spread across a large number of electrodes however, this rhythm may be attenuated when using a common average reference. Thus, the benefit of using data-driven spatial filters is that they may work in both cases~~. In addition, using spatial patterns as estimated from the covariance matrix between channels may be helpful as a tool for data exploration to evaluate this factor~~, because the spatial correlations across electrodes for different present rhythms cannot be known a priori.

And extended the the discussion regarding simulating a ground truth (page 25, line 804):

As a general limitation, the estimation of a backward model will never achieve perfect accuracy because dozens of electrodes are not enough to capture the thousands of underlying sources of neuronal activity. One approach for addressing this would be through incorporating simulated iEEG data, where the ground truth is known, such as the LFPy toolbox (Hagen et al., 2018). Specific limitations of an approach for estimating spatial filters utilizing eigenvalue decomposition are detailed below.

For Section 3.4, would adding a figure showing the SNR distribution (w/t thresholding) for some components within predefined frequency bands (delta/theta/lower beta) be useful to further emphasize the point that a small set of subjects can drive overall results? If the SNR distribution has a long tail, this would be evidence in favor of this hypothesis.

As per suggestion, we also tried to incorporate visualizating SNR by binning according to fixed bands.

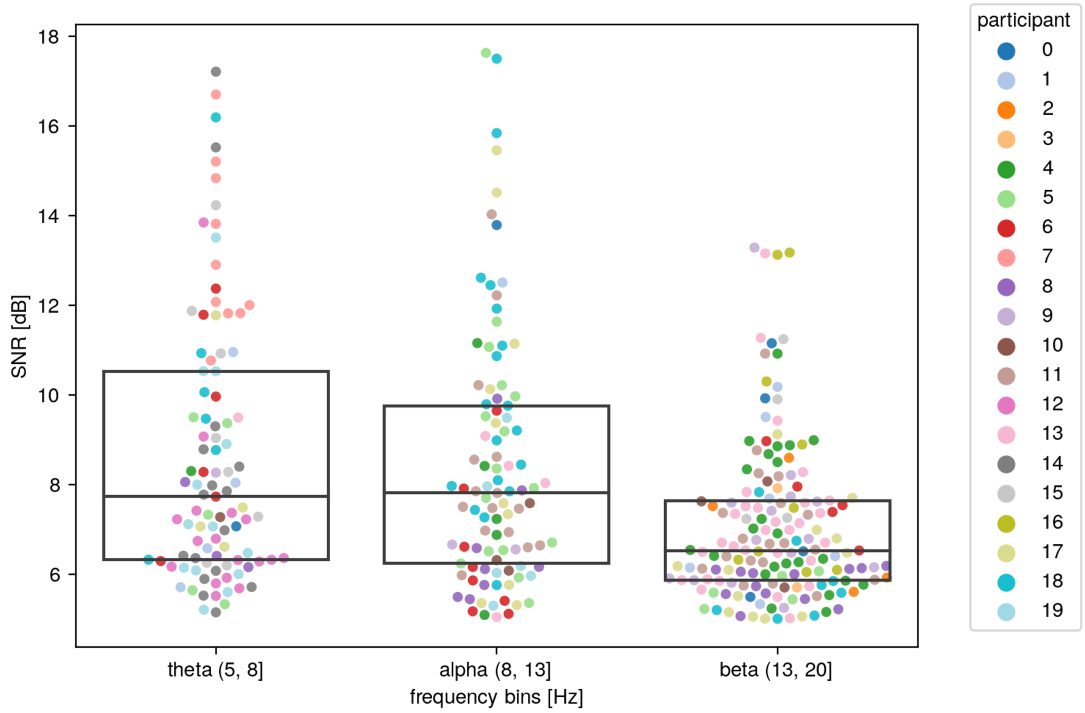

The individual points constitute components for the respective color-coded subjects. It can be seen that the distribution has a long tail (especially considering that the y-axis is in log-scale) and some participants dominate the upper tails. As we generally advocate against binning based on fixed frequency bands, we find such a plot problematic, also given that all these components originate from very different cortical regions. We therefore did not include this plot in the manuscript.

R1 comment #5

1. Line 37, ‘used here are’ -> ‘used are’

First fixed, but then this sentence was scraped. :)

R1 comment #6

2. In lines 195/196, it is quite plausible for the covariance matrix to be not full rank here, so is there some regularization applied? In the code I see that it says it

expands the matrix in this situation, expressing that in the Methods in an equation/a line seems important.

The reviewer is right, In the case of the covariance matrix not having full rank, the matrix is expanded. We have added this paragraph in the methods (page 9, line 293):

A technical note: certain preprocessing operations, like removal of ICA components, can result in a covariance matrix that does not have full rank. To determine whether the covariance matrices have full rank, the eigenvalue problem involving only the signal covariance $C_s V = V\Lambda$ is solved. The rank *r* is determined by calculating the number of eigenvalues that are not zero (above a small numerical threshold, 10^{-6}. If the matrix is not full rank, SSD is computed on the expansion: $\widetilde{C}_s = (V_{1:r}\Lambda)^T C_S V_{1:r}\Lambda$ V_(1:r) Λ, with Λ being the identity matrix, having $1/\sqrt{\lambda_i}$ on the diagonal, with eigenvalues λ_i and $V_{1:r}$ constituting the first *r* eigenvectors. After solving the generalized eigenvalue problem, the resulting spatial filters $\widetilde{W}$ are multiplied with $V_{1:r}\Lambda$ to obtain the spatial filters in the original space: $\widetilde{W} = V_{1:r}\Lambda W$.

R1 comment #7

3. In line 217, what exactly is being inverted here, C*W?

We apologize for being unclear here and expanded the description methods to accurately reflect the calculation of the spatial patterns, the spatial patterns A can be calculated in two ways, either by inverting the spatial filter matrix $A=W^{-1}$ or by multiplying the spatial filters with the covariance matrix. As we do the latter in the code, we now describe this method in the method section. We thank the reviewer for this comment, because we feel that this aspect is now much clearer, and the formulas appearing in the illustration Fig. 1 are also now also included in the main text, which should make the approach more accessible (page 9, line 317):

Spatial patterns $A$for interpretation of the spatial origin of the extracted component can also be obtained ~~are obtained with aid of covariance matrices~~ by multiplication of spatial filter matrix W with the covariance matrix calculated for the signal component in the frequency band of interest ~~and the pseudo-inverse of the spatial filters~~ C_S (Haufe et al., 2014b): $A = \frac{1}{Z} W^T C_S$. For appropriate scaling, the patterns are normalized by a scaling factor $Z = (W^T C_S)^+ W$, with $^+$ denoting the Moore-Penrose pseudoinverse, such that the product of spatial patterns and spatial filters will yield the identity matrix

$A^T W = Id$. This is required in order for the product of the patterns and source estimates $\hat{S}$ to yield the electrode measurements X.

R1 comment #8

> In lines 220 to 222, it's unclear why a subset of electrodes based on a distance metric from the maximum location are chosen.

The idea of choosing a subset of electrodes based on the distance was to quantify the spatial spread fall-off using a Gaussian 'full width at half maximum'-type metric. In the cited simulation work by Muller et al. 2016 the fall-off was modelled with different types of functions. Because the spatial sampling in our used dataset is coarser (10 mm spacing between electrodes) compared to the high density-grids used by Muller et al (4 mm spacing), we did not perform a function fit of the correlation profile / spatial pattern coefficients and opted for a simple metric here.

- L. Muller, L. S. Hamilton, E. Edwards, K. E. Bouchard, E. F. Chang, Spatial resolution dependence on spectral frequency in human speech cortex electrocorticography, Journal of Neural Engineering 13 (5) (2016) 056013.

We clarified this aspect by extending the methods section (page 9, line 327):

> To illustrate spatial spread of oscillatory components, we analysed the topography of spatial pattern coefficients. For each component, the absolute value of the associated spatial pattern coefficients was taken and the values were then divided by the maximum value. The maximum spatial pattern coefficient in a distance of 2.5 cm around the maximum (distance value determined by Euclidean distance) was extracted to assess contribution of a single component onto several electrode signals. We chose to limit the calculation to the immediate surrounding of the spatial maximum based on work from Muller et al. (2016), who modelled the decrease in spatial correlation across electrodes using different function fits in high density ECoG data. As the spacing across electrodes in the dataset used here was too coarse to fit a function, we opted to quantify the decrease in spatial spread across space by the maximum spatial pattern coefficient in the vicinity of the spatial maximum.

R1 comment #9

Line 255, what is the band range used to define the noise?

The used band range is the desired peak frequency ± 1.75 Hz for defining the maximum SNR component (= the component that is set to be removed, e.g. constituting line noise). For the contribution that should remain in the data, we used a lower frequency band value of 1 Hz, so that the activity over the whole frequency range would be used for calculation of the corresponding covariance matrix. We apologize for the oversight to describe this more detailed in the methods and have updated the description in the methods for clarifying this and also highlighting the flexibility of SSD in that respect (page 10, line 359).

> For removing noise with a specific spectral profile, we estimate spatial filters for maximizing SNR around the frequency peak that should be removed, e.g., 60 Hz ± 1.75 Hz for line noise. ~~Then these components~~ For defining the contribution that should be minimized, i.e. the contribution that should remain in the cleaned data, we adjusted the used frequency ranges slightly: while previously only a narrow frequency range was used for defining the flanking frequency, here we adjusted the lower range of the flanking pass-band to be at 1 Hz, such that the activity across the whole frequency range should be considered to remain in the data. The adjustment of frequency borders is a benefit of SSD, as it allows for flexible incorporation of prior knowledge for estimation of spatial filters. After estimation of spatial filters, the components constituting line noise are subtracted from the raw signal with a linear operation:

R1 comment #10

Line 295: ‘which utility’ -> ‘whose utility’

Fixed, thank you!

R1 comment #11

Lines 310-311: ‘Using SSD results in less bias’ -> Bias in a statistical sense refers to a difference from the expected mean, so I was unsure how it was being used here. I do agree that SSD makes fewer assumptions about the nature of how sources are distributed/how they are getting mixed and so this terminology might be better fit or perhaps just a definition of what is intended in using the word ‘bias’.

Thank you for this comment, this helped us to clarify terminology. We have updated this paragraph to reflect our intention when using this term (same paragraph as in comment R1 comment #4, page 12, line 450):

> Additionally, all signals were submitted simultaneously to the SSD procedure, without subselection, making it possible to combine information from multiple leads and electrode configurations efficiently. ~~Using SSD results in less bias due to fixed reference choice, as learning the filter coefficients from the data enables recovery of rhythms in a flexible way regardless of their location and dipole orientation.~~ While standard referencing techniques tend to enhance signals generated via a specific way, SSD is agnostic to the biophysical generation, and can be used more flexibly in this regard. For instance, monopolar and common-average referencing preserve correlation across channels to a higher degree than bipolar referencing (Li et al., 2018). This will influence measurement of rhythms, which can have a different spatial spread across subjects, depending on local cortex anatomy. So, while common average referencing highlights radial sources, bipolar referencing has a focus on locally generated activity and emphasizes bipolar sources. SSD will extract components with maximum SNR agnostic about their spatial spread. In the next section, we further examine the spatial spread present empirically in iEEG data.

R1 comment #12

> While a paragraph in the results is dedicated to discussing the possibly ways we could apply statistical tests to SSD in order to perform inference on the components (lines 343 - 347), why isn't it implemented here? I suppose SSD is being demonstrated here as a data exploration tool, in that case this being the reason no statistical tests are implemented needs to be made explicit in this section or earlier in the Methods.

The reviewer is right that our main focus here is to promote the usage of SSD as a data exploration tool. In our view, statistical testing would be highly dependent on the specific objectives one has, and there may be different criteria required. We now also provide the eigenvalues as a return argument, which correspond to the local relative SNR-threshold criterion as in Nikulin et al., 2011. Personally, we have not used this criterion, as we prefer an explicit 1/f-correction. We extended the paragraph regarding this, page 15, line 502:

> The determination of which components to keep can be made using several different approaches, such as a threshold criterion based on ~~relative SNR~~1/f-corrected SNR as in this article, a more local relative SNR-threshold criterion only focusing on the peak frequency band and neighboring flanking bands (Nikulin et al., 2011; Haufe et al.,

2014a), with the aid of a bootstrapping procedure (Zuure et al., 2020), or based on physiological considerations such as focusing on rhythms originating from a specific location, which can be determined with aid of the spatial patterns. We want to stress that a criterion of the number of components to keep is dependent on the specific objectives of the study and needs to be carefully considered within the scope of those desired objectives. In the following, we employed a 1/f-corrected SNR criterion, as the aim was to quantify all dominant resting rhythms without any regional pre-selection.

R1 comment #13

9. Line 353: what threshold of SNR was applied? 5 dB?

Yes, we added this description also here, page 15, line 519 (in addition to the existing text in methods).

The components with SNR exceeding a specified threshold (>5 dB) were retained.

R1 comment #14

10. Lines 370-371: I understand this is of concern because of the possible effects of a few outliers on results, but that needs to be made explicit in this line.

Thank you for pointing this out, it's important for us to get this point across and we have therefore expanded this section with additional details, page 17, line 538.

Another factor to consider, especially in iEEG data, is the fact that patients have the grids implanted for clinical reasons, with different pathologies and different medication status, which might contribute to the observed variability. Nevertheless, variability in peak frequencies and oscillatory SNR is also observed in non-invasive electrophysiological measurements. For iEEG, this might be more of a concern, since a smaller number of participants are usually included per study, compared to studies using non-invasive measurements. In the case of such small sample sizes, a single participant with a large amplitude, prominent rhythm across many electrodes may dominate the analysis due to the way that iEEG data are often pooled. This can result in a seemingly large effect in the group-average, despite only being present in a small number of participants.

R1 comment #15

11. In Figure 5, I was unclear what is meant when there's no sphere in 5A, no dominant frequency present at that electrode?

Correct, in that case, there is no component of that dominant frequency (= above the SNR-threshold) with a spatial maximum over that particular location. We extended the description in the legend to clarify this.

> Figure 5: A) Each subplot shows the location of electrodes (white squares) on a template brain for one individual participant. Each sphere indicates an oscillatory component, with the size indicating 1/f-corrected SNR and the color indicating peak frequency of that component. If there is no sphere of a respective color in the vicinity of an electrode, no rhythm above the SNR-threshold could be detected in that frequency band. There is large variability between participants. For improved comparison across participants, all electrodes and rhythm locations were mapped onto the right hemisphere. Participants are ordered according to the mean z-coordinate across the electrode grid, to ease comparison.

R1 comment #16

12. In Figure 5B how is it possible for multiple components to have peak frequencies so close to one another? i.e. don't the bandpass filters overlap considerably for some of these components?

Two key aspects are related to this comment:

1. The peak frequency that is used as the SSD parameter does not necessarily exactly correspond exactly to the peak frequency of the resulting components. So, while the peak frequency lies in the used passband for defining the signal, there is some variation in peak frequencies, for instance when using a passband of 8-12 Hz, it is possible that one component could have a peak frequency of 8.5 Hz and another 11.5 Hz. This will result in slightly varying colors in Fig. 5B. But typically the range of peak frequencies is closer together for the top SNR components.
2. Sometimes, there are peak frequencies in the raw signal that are quite close by. In that case, for Fig. 5B we ran SSD for all of these frequencies that exhibited a prominent peak in the electrode signals, the computation was performed separately per frequency. For instance, in the figure below we show power spectra, topographies and a time series extract for components with a peak frequency of 9.33 Hz and 7.00 Hz that are neighboring in peak frequency and location for the participant from Fig. 2 of the main manuscript.

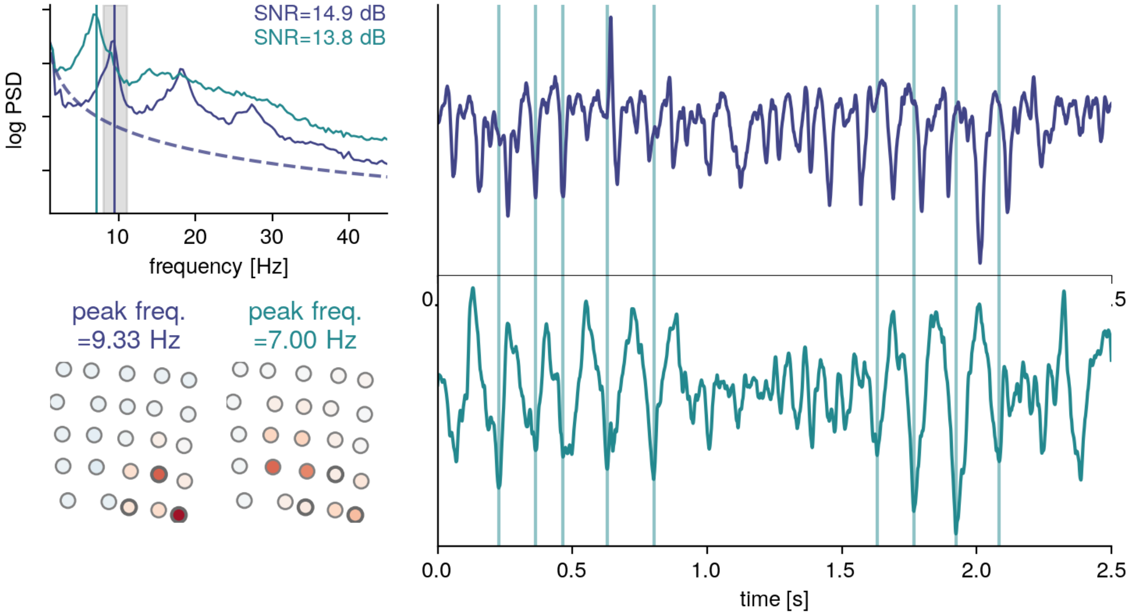

To clarify the first point, in addition to the already stated motivation for this procedure in the methods section, we have extended the description of Fig. 5B in the results section (page 17, line 527):

> We also show the peak frequency of all identified components in 5B and C. We find a distribution similar to Groppe et al. (2013), where there are more rhythms detected with a peak frequency around 7 as well as 16 Hz, and fewer rhythms with a peak frequency around 10 Hz, in contrast to non-invasive electrophysiological measurements. Note that because we use spectral parametrization on the spectra of SSD components, the peak frequency of SSD components can slightly vary from the peak frequency used as an input parameter for SSD. The distribution of peak frequencies is possibly related to the spatial bias of electrode placement, wherein most are placed over sensoriomotor and temporal areas, with less coverage over occipital areas, as determined by clinical needs.

R1 comment #17

13. Line 404: ‘am’ -> a

Fixed, thanks!

R1 comment #18

> 14. Line 450, same question about bias as in Q7, also I think there is one other time bias is used this way.

Please see our reply to the above comment R1#11 for this.

> I hope the comments are helpful and want to reiterate that I think this is valid and useful contribution.

We are grateful to receive so many thoughtful comments and had fun thinking about the raised issues!

Reviewer #2:

In their manuscript entitled "Enhancing oscillations in intracranial elecrophysiological recordings with data-driven spatial filters" the authors Schaworonkow and Voytek report a useful new method and demonstrate its application to a number of datasets. The paper is clearly written, well justified, and the results support the position that this represents a useful tool. I thank the authors for using publically available data and making their code available. I have a few comments in order below that might improve the paper, and believe that it is well suited for publication.

> I cloned the repo and but was unable to get the code to run, so I will not comment on it specifically, with the following error. [ERROR]

We apologize for this oversight! The problem was related to a used toolbox, we have now provided a code version where this type of problem is avoided. Additionally we checked that the code runs on another machine with a fresh checkout of the repo.

R2 comment #1

> 1) MAJOR. I was a bit confused about the distinction between electrocoritcography, intracranial recordings, and iEEG, and I think later sEEG. I think this paper is focused on electrocorticography (on the surface of cortex), as opposed to depth electrode arrays (intracranial depth electrodes). I think it would help the reader to clarify this early on and

> in the discussion perhaps indicate how these methods might apply to linear arrays of depth electrodes.

We abbreviate intracranial recordings with iEEG. In our manuscript we distinguish two types of iEEG recordings, electrocorticography (ECoG) using electrode grids placed on the surface (like in Fig. 2) and stereoencephalography (sEEG) using electrode strips (like in Fig. 3). We mostly use ECoG, because that's what is the type of electrodes in the majority of the dataset we use.

To clarify this, we extended the paragraph in the introduction (page 2, line 2):

> Invasive, intracranial electroencephalography (iEEG) recordings from patients undergoing epilepsy monitoring have been tremendously valuable for examining neuronal activity. This is because iEEG provides both high temporal and spatial resolution that is impossible to achieve using solely noninvasive human neuroimaging (Engel et al., 2005; Jacobs and Kahana, 2010). <u>There are different types of recording electrodes for iEEG, using electrodes arranged in grids that are commonly referred to as electrocorticography (ECoG) or using electrodes arranged along a linear array, which is referred to as stereoencephalography (sEEG).</u> Because of the superior spatial and temporal resolution of iEEG, combined with the possibility of simultaneous single-neuron recordings from humans (Suthana and Fried, 2012), these rare recordings provide a bridge between human cognition and decades of animal electrophysiology.

Additionally, we extended a paragraph in the discussion section (page 22, line 656):

> The benefits of using statistical approaches like SSD, in contrast to biophysical modelling, is that no anatomical information or biophysical model is required for the estimation of the spatial filters, which strongly reduces the complexity of the procedure. <u>While our demonstrations are mostly using ECoG data (as this was the predominant recording type present in the used dataset), the method can be similarly applied to sEEG data, as seen in Fig. 3, for the benefit of combining information from different electrode leads.</u> Whenever time series data from multiple electrodes is available, the method can be applied. The electrode locations are only needed for the interpretation of spatial patterns, but the source time series estimation is independent from the localization accuracy of the electrode positions.

R2 comment #2

> 2) MAJOR. Independence of components. I was hoping for some discussion of the relative orthogonality and independence of the components obtained by this method as opposed to simple eigenvalue decomposition, ICA or PCA. No mention or rotation for orthogonality, etc. Are the components obtained independent of one another. The text is often written as if they are (multiple oscillatory sources from same coritical area). Can

the authors show mathematically or experimentally how independent their components are and if this is desired.

We extended the description in the methods sections at page 8, line 277. Please also see the next comment #3 for more details the difference between spatial filters and patterns.

The number of components returned by SSD is equal to the number of electrodes, with the components ordered by relative SNR in the frequency band of interest. In contrast to PCA, the first few SSD components only capture a small fraction of global variance, as the method is focused on maximizing variance in a specific frequency band. PCA has strong constraints, and can only return a spatial filter matrix W which is orthogonal, i.e., for which $W^T W = Id$ needs to be satisfied. Therefore $W^T = W^{-1} = A$, which means that spatial patterns A are equal to spatial filters for PCA. As this results in spatial filters with high degree of smoothness between neighboring values, PCA will not be able to distinguish rhythms in the same subspace. SSD and other generalized eigenvalue decomposition methods do not have this constraint. There, the following constraint needs to be satisfied $A^T W = Id$, so the spatial patterns are not generally equal to the spatial filters, as the inverse of a matrix is not generally equal to its transpose. This allows distinguishing sources in the same subspace, e.g. rhythms coming from the same cortical area in the same frequency band. In terms of amplitude of the individual components, they are independent of each other for PCA as well as SSD.

R2 comment #3

3) I found most of the math intuitive except the difference between spatial filters and patterns. It seems strange they are different at first glance. I think this is just because the filters are the linear combo if signals needed to derive a source from the data itself. Perhaps a bit more prose regarding your intuitions for why these are different and that is expected would help?

For making the difference between the two concepts clearer, we have included the associated formulas in the methods, page 8, line 267:

While the spatial filters are estimated with the aid of covariance matrices obtained from narrowband activity (from the narrowband activity defined as signal as well as the flanking narrowband noise), the spatial filters are then applied on the broadband ~~signal. The application to a broadband signal~~ activity recorded by the electrodes X to yield the component time series $\hat{S} = W^T X$. The data can be reconstructed using $X = W^{-1}\hat{S} = A\hat{S}$, where the inverse of the spatial filter matrix W^{-1} constitutes the matrix of spatial patterns A.

Additionally, we have modified the Introduction, where we first introduce the difference between spatial filters and patterns, as follows (page 5, line 100):

> It is important to make a distinction between spatial filters and the spatial patterns associated with each filter. A spatial filter assigns a weight to each electrode that quantifies how much each electrode contributes to the calculation of an extracted component. A spatial filter is generally not interpretable (Haufe et al., 2014b), however, in the sense that the magnitude of the weights directly reflects the contribution of the source to the spatially filtered signal. This information about spatial origin of a component can be found in the spatial pattern, which can be computed for each spatial filter and reflects , as a large spatial filter weight may also be related to cancellation of noise, for instance. Once the spatial filter weights are calculated, one can examine the spatial structure of each component by computing the spatial patterns, with each pattern reflecting the mapping of sources onto measured electrode signals, showing. This quantifies the strength and polarity of a putative source signal on all electrodes. For instance, in Fig. 1, for the bipolar referencing only two electrodes contribute to the calculation of the component. But due to the fact that neighboring electrodes exhibit signal correlation to the involved electrodes due to spatial spread, information about this source is also present in the vicinity of the two electrodes used for calculation of the bipolar derivation. Therefore, the associated spatial pattern has intermediate coefficients around the involved electrodes. The spatial patterns are can for instance be computed by matrix inversion of the spatial filters. It can be seen that although the spatial filters in Fig. 1 have different structure respectively, the associated spatial patterns are quite similar, reflecting a source originating in the sensorimotor region.

R2 comment #4

> 4) Remove artifacts without artifacts from temporal bandpass filtering. I find this hard to buy, seems to good to be true. Maybe this needs to be watered down a bit. Of course one source of "artifact" or a-perfection in the filtering is the width of the band used in the covariance matrix computation (attentuation just outside the filter band). I might argue the imperfection of the filter used to construct the narrow band signal matrix also would introduce analagous artifacts. Perhaps these statements can be tempered a bit.

The reviewer is right that removing noise always comes with a cost. Here, we would say that the main risk is removing information that is not line noise / interesting signal contributions. That can happen when too many line noise components are removed, in an attempt to reduce the line noise. This is especially problematic when noise and signal contributions cannot be reliably separated. Please also see R1 comment #9 for a clarification on which frequency bands were used to calculate SSD filters for removing line noise.

We extended the paragraph (page 19, line 622) to be clearer in this respect:

> The cost of this type of noise removal is the loss of dimensionality equal to the number of removed components, similar to the effect of applying a common average spatial filter. This loss of dimensionality is not of concern when a high number of electrodes are present, but would not be recommended for a small number of electrodes. While a common average spatial filter may work well if there is a common noise source that is manifesting in all electrode signals, using data-driven spatial filters allows for more flexibility if noise is not present in all signals. If too many noise-related components are removed, this bears the risk of removing signal contribution that is of interest to the research question. It is therefore necessary to inspect the estimated noise components, for instance regarding the presence of spectral peaks in the frequency band of interest. If time-locked analyses are performed, noise components can also be inspected for the presence of time-locked contributions, to rule out reduction in valuable information by noise component subtraction.

R2 comment #5

> 1) bottom of page six, indicate the covariance matrix are over channels (says it later, but useful here)

Good point for clarification, we included this at page 8, line 251.

> The covariance matrices across electrodes of the signal and noise contributions are calculated on the basis of the band-pass filtered electrode activity.

R2 comment #6

> 2) pg. 7 "while the spatial filters are estimated with the aid of covarianc ematrices obtained from narrowband activity, ..." this confused me on first read since they were also made with the braodband noise activity just as much, probably rephrase.

The spatial filters are also constructed with noise contribution, but this is also of narrow-band nature (this is specifically for SSD, variants like Mike Cohen's GEDb method use broadband noise). We included a statement clarifying that on page 8, at line 267:

> While the spatial filters are estimated with the aid of covariance matrices obtained from narrowband activity (from the narrowband activity defined as signal as well as the flanking narrowband noise), the spatial filters are then applied on the broadband ~~signal. The application to a broadband signal~~ activity recorded by the electrodes [...]

And on page 8, line 237:

> We estimate spatial filters via spatio-spectral decomposition (SSD) (Nikulin et al. 2011), which specifically maximizes spectral power in a frequency band of interest (for example from 8–12 Hz), while minimizing spectral power in flanking frequency bands (for example from 6-7 Hz as well as 13-14 Hz). This procedure enhances the height of spectral peaks over the 1/f-contribution, exploiting specifically the typical narrowband peak structure of neural oscillations.

R2 comment #7

> 3) OUT THERE. One connection my brain made is that accentuating the size of effects in this way might introduce a dangerous potential to find "voodoo correlations". In that old Vul work, they showed that "using a strategy that computes separate correlations for individual voxels, and reports means of just the subset of voxels exceeding chosen thresholds. We show how this non-independent analysis grossly inflates correlations". Is there a danger of the field over estimating effect sizes if we accentuate the size of the effects in the way proposed here, and what steps should researchers take to avoid this pitfall?

The reviewer makes an important general point. We think whether preprocessing techniques that increase SNR can lead to circular analysis depend on the analyses that are performed afterwards. For instance, we deliberately did not include a statistical test regarding SNR of SSD compared to other reference choices, as this would fall into this sphere of performing circular analysis. In general, many procedures strive to increase SNR (e.g., source reconstruction, standard reference schemes aiming to reduce common noise or emphasize activity coming from different areas, or even simple trial-averaging). In that respect, we see SSD and other spatial filtering techniques as one tool in the toolkit for researchers interested in specifically oscillations.

This note relates to our way of thresholding components based on SNR for the inter-individual variability analysis. We therefore now included a cautionary note on this topic in the discussion, page 25, line 826:

> In deciding how many components to keep for analysis, the following aspects should be considered when using SSD: Inspecting the relative SNR with aid of the power spectrum is crucial and is simplified because the components are ordered according to SNR in the frequency band of interest. Components without a spectral peak in the frequency band of interest should not be considered when talking about neural oscillations in that specific frequency band (Donoghue et al., 2021). The spatial patterns

should be inspected for determining the local focus of the generating source. In addition, bootstrapping approaches based on surrogate data have been suggested to estimate the number of components to retain (Zuure et al., 2020). Regarding the accuracy of reconstruction for instance in the example in Fig. 2, it can be seen that the spatial focus lies on the edge of the recording electrode grid. In that way, the quality of reconstruction is limited by not having more electrodes bordering the spatial maxima. In general, optimizing for SNR and then checking for the presence of high SNR bears the risk of circular analysis. Here, we use a moderate SNR-threshold to retain components. Because our focus on these results is to highlight the degree of variability in the individual recordings, we did not perform a bootstrapping analysis. However, if we wanted to quantify whether the component structure contains more oscillatory structure than expected when running the analysis using spatially correlated 1/f-activity contributions not containing oscillatory bursts, bootstrapping would be appropriate. Circular analysis is not of concern when relative contrasts within conditions are computed, for instance across trials for one participant, where the SSD spatial filters were estimated on the whole data segment, because in this case it is not the absolute oscillatory power that is crucial, but rather consistent power in- or decreases across experimental conditions.

And include a guiding note at the beginning of the discussion, page 21, line 636:

In this article, we highlighted the benefits of using spatial filters for the extraction of neural oscillations in invasive electrophysiological recordings. Applying spatial filters that specifically optimize for oscillatory SNR in iEEG recordings, we assessed presence, spatial spread, variability and waveform shape of iEEG resting rhythms. SSD and other spatial filtering techniques can be a potential tool in the toolkit for researchers specifically interested in oscillations. As with all tools, careful consideration of the benefits and limitations has to be weighed against the increased complexity and freedom in parameter choices that might give way to potential false positives.

R2 comment #8

4) Researcher degrees of freedom - I tend to avoid any component selecting in my analysis pipeline, as Laszlo showed that ICA component selection success varries with experiementer experience. Can you imagine more automated ways of selecting components than the heurestics proposed in the paper?

We agree with the reviewer that the selection of components introduces researcher bias. In contrast to ICA, the benefit of SSD is that the components are ordered according to SNR, so it's possible to define a cut-off criterion, without inspection of all individual components. In most of our previous studies using SSD we used a 1/f-corrected SNR criterion of 5 dB like in

the present manuscript, which for us constitutes a basic oscillation presence check. In that way, a cut-off criterion constitutes at least an improvement over inspection. For modifications of manuscript text regarding this aspect, please compare to R1 comment #12 (pasted below for convenience, from page 15, line 502) ,where we stress that we use SSD as a tool for data exploration, so the criterion which components are to be selected may also be informed by the study objectives, for example if rhythms of a specific location are of interest.

> The determination of which components to keep can be made using several different approaches, such as a threshold criterion based on ~~relative SNR~~1/f-corrected SNR as in this article, a more local relative SNR-threshold criterion only focusing on the peak frequency band and neighboring flanking bands (Nikulin et al., 2011; Haufe et al., 2014a), with the aid of a bootstrapping procedure (Zuure et al., 2020), or based on physiological considerations such as focusing on rhythms originating from a specific location, which can be determined with aid of the spatial patterns. We want to stress that a criterion of the number of components to keep is dependent on the specific objectives of the study and needs to be carefully considered within the scope of those desired objectives. In the following, we employed a 1/f-corrected SNR criterion, as the aim was to quantify all dominant resting rhythms without any regional pre-selection.

R2 comment #9

> 1) Pg. 15, ln 404, typo "am"

Fixed, thanks!

R2 comment #10

> 2) Figure 6 - I think e1 and e2 should. be above the comp 1 and 2 to match other figures

Thank you, we adjusted the figure according to your comment for increased consistency.

R2 comment #11

> 3) Figure 7 - can you show a panel here using a normal bandpass noise filter that many people would use, to show the bleed over into neighbouring bands. I think for Figure 7 adding a panel D with a narrow band 60 Hz filter will show how much better yours is.

According to the suggestion, we added a panel with a standard notch filter from MNE, using the noise peak frequencies, see the updated Fig. 7. The notch filter was applied on the

common-average referenced data, since we think that would be the most typical workflow for iEEG processing. The artefacts in the spectrum are clearly visible.

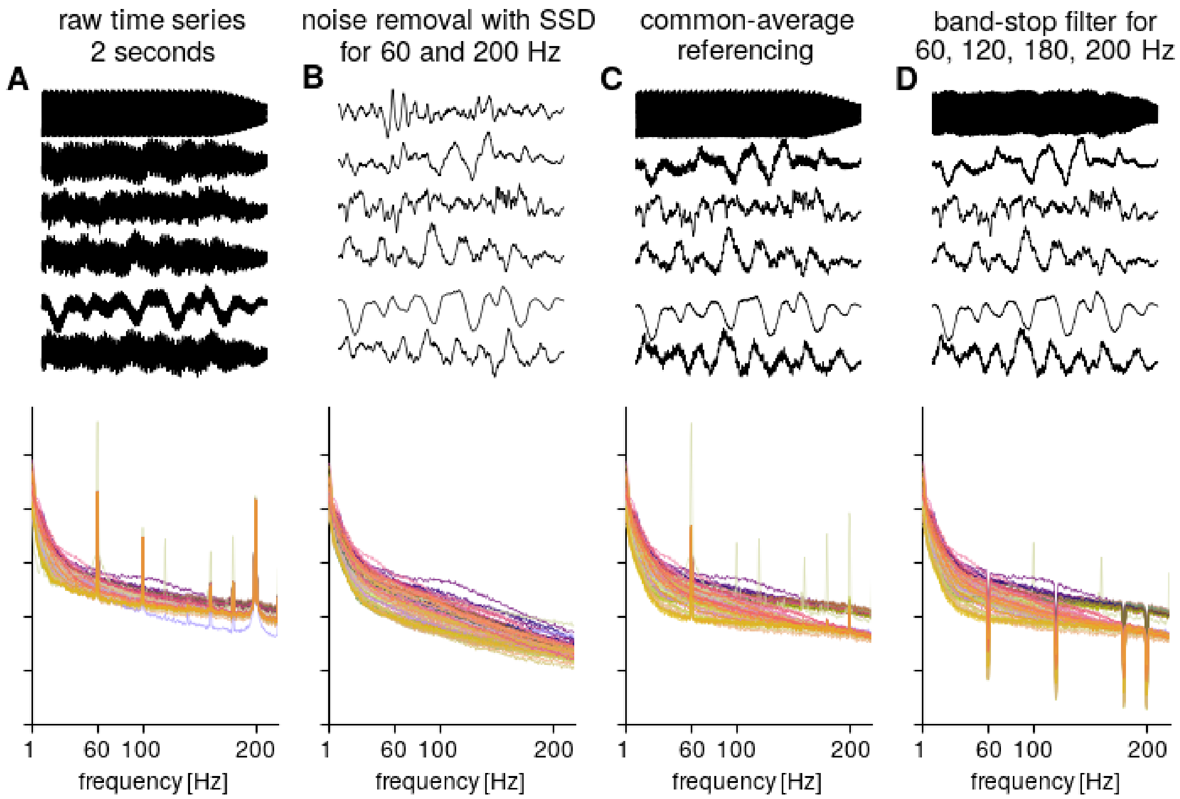

A) Time series of six electrodes and power spectra for raw ECoG recording for 87 electrodes, color code corresponds to electrode position, with neighboring electrodes having a similar color. B) Time series and power spectra after removal of components maximizing SNR for 60 Hz and 200 Hz spectral peaks. Note that there are no band-stop type artefacts in the spectrum since no temporal filtering was performed. C) Time series and power spectra after common average referencing. While the 200 Hz noise is largely attenuated, 60 Hz line noise still persists. D) Time series and power spectra after common average referencing and then band-stop filtering. The band-stop filters introduce artefacts in the spectral domain.

R2 comment #12

4) Waveform shape section - I like this and you did show that it was working well after the transformation, but if the hypothesis of this paper is "this technique works better than others" and this section is "it also works for bycycle analysis", then you should probably compare the results to those obtained from non-transformed data.

We tried to state the benefit of the approach as more flexible, not necessarily as better, because “better” depends on the respective criteria used for evaluation. Please compare the reply and text changes to the related comment #4 from reviewer 1. Specifically for waveforms, the difficulty in comparing the time series results for waveforms is the choice of comparison electrode, which can show a mixture of the waveforms present as shown in Fig. 6A. Such a comparison may also delve into circular analysis, because all waveform shape phenomena are highly dependent on SNR; we therefore have deliberately left that out. Our main point here in Fig. 6D & E was simple to show that non-sinusoidal waveforms are present throughout the cortex and may be an interesting feature to look at in ECoG studies as they allow access to a higher SNR and therefore detectability of these phenomena.

R2 comment #13

> 1) limitations - This section was good but was a bit detached from YOUR analysis and results. Can you point to a couple of your results in each of these limitations. When you say backwards model doesn't "acheive perfect accuracy" - what does this look like in the data, bleed over into components, not separating them well, etc. For the travelling wave example, could this explain any of your results in a different way (multiple sources of alpha from one location)

This comment is tricky, as these things are hard to substantiate without a known ground truth. See also related R1 comment #4. We extended the limitation section by referencing our Fig. 2 example participant to make the raised points more easy to grasp with examples.

Regarding the first listed limitation about accuracy of source reconstruction, page 25, line 831:

> The spatial patterns should be inspected for determining the local focus of the generating source. In addition, bootstrapping approaches based on surrogate data have been suggested to estimate the number of components to retain (Zuure et al., 2020). Regarding the accuracy of reconstruction for instance in the example in Fig. 2, it can be seen that the spatial focus lies on the edge of the recording electrode grid. In that way, the quality of reconstruction is limited by not having more electrodes bordering the spatial maxima.

Regarding the second listed limitation about signal polarity, line 849:

> Further, the estimated spatial filters are invariant with respect to signal polarity, i.e., the sign cannot be uniquely determined. Therefore depending on the choice of parameters, the spatial filter can result in a polarity-inverted signal. For instance, for the participant in Fig. 2, the time series and spatial pattern of the second and third component was manually multiplied with -1 for visualization. Alignment of spatially filtered signals can

for instance be accomplished according to the sign of the electrode signals, and is straightforward in the case of radially orientated components.

Regarding the 3rd listed limitation about linearity & waveform shape, line 860:

Finally, the underlying assumption here is a linear model, and the estimated spatial filters are not dependent on time. This assumption might insufficiently capture traveling wave phenomena, for instance. Propagating activity with high velocity will impact very sharp waveforms, as for electrodes linearly combined with a slight offset a sharp trough will result in a less sharp trough for the component due to time-independent linear combination. For instance, the waveforms in Fig. 2 will display a higher peak-trough asymmetry when calculated on high-SNR segments directly from the electrodes, while SSD component traces will have a slightly lower asymmetry measure due to the spatial filtered signal being a linear combination of slightly time-shifted oscillation. It would be of interest for future directions to take wave propagation into account when estimating neuronal oscillatory sources (Kuznetsova et al., 2020; Hindriks, 2020).

We thank the reviewer for all comments and hope to have clarified the raised concerns regarding properties of SSD components, the difference between spatial filters and spatial patterns and further aspects of the noise removal via spatial filters.
