# Supplementary material for: Enhancing oscillations in intracranial electrophysiological recordings with data-driven spatial filters
Source: PLoS Comput Biol. 2021 Aug 19;17(8):e1009298. doi: 10.1371/journal.pcbi.1009298 (PMC8407590; doi:10.1371/journal.pcbi.1009298)
Supplement: Attachment — Submitted filename: Enhancing oscillations in intracranial electrophysiological recordings with data.docx [file pcbi.1009298.s003.docx]

Enhancing oscillations in intracranial electrophysiological recordings with data-driven spatial filters

This study makes a great case for using spatio-spectral decomposition as a data-driven referencing technique for iEEG as opposed to using methods such as bipolar or Laplacian or common-average referencing. The data-driven nature of this strategy is clearly to its benefit and can be incredibly relevant when signals tend to be spread simultaneously across multiple electrodes, for example, due to volume conduction. While I have been convinced of the value of this method and its usefulness for application to iEEG data, I found aspects of the narrative difficult to follow and several conclusions to be too broad.

**Larger scale issues:**

1. In the Introduction, the paragraph beginning at line 29 appeared to jump between spatial filtering and source reconstruction. I think reorganizing it in a way that highlights how source reconstruction is also a form of spatial filtering will help make it clearer (maybe):
   a. source reconstruction can be reframed as a spatial filtering problem that depends upon several assumptions
   b. past work has built numerous methods that fit into this mold (biophysical modeling, ICA, etc.)
   c. referencing is a particular instance of spatial filtering, and the common referencing strategies used, while appearing to have no assumptions makes strong assumptions about the nature of the sources measured
2. Referencing has a clear physical basis: that we are estimating voltage gradients. Note also in source reconstruction we are identifying dipole sources, so that the ‘filter’ applied there leads to clear physical intuition about the outcome. As such, I felt confusion about the physical intuition offered by application of SSD due to the confounding of (i) spatial mixing of multiple independent sources due to volume conduction with (ii) multiple, independent sources all coordinated at/near zero phase. I don’t think the two cases can be separated under SSD unless the sources have unique waveforms/unique sources of noise or the results under a Laplacian/Bipolar referencing scheme are compared to SSD. If there is no consistent rhythmic activity in the Laplacian/bipolar but we see weights across multiple electrodes in the SSD this is evidence suggesting that there is a common source.
   a. Lines 46-47: While the idea of spatial superposition of multiple rhythms is relevant to when multiple independent (potentially coherent) rhythms contribute to multiple electrodes, spatial superposition needs to be differentiated from the idea that any single low frequency band contributes to multiple electrodes due to volume conduction (which could equally well happen at low and high frequency bands). The high correlation of low frequency bands may well be due to independent sources becoming coherent rather than a single source that shows up at multiple electrodes. This interpretation is present in lines 77-79, While the first part of the line speaks to rhythms becoming coherent over some spatial area, the second part (‘spatial mixing’) seems to be speaking to when volume conduction plays a part in what electrodes record, but it is spoken of as though they are the same phenomenon.
   b. Lines 58-59: I think the expectation that SSD extracts ‘distinct neural sources’ is not borne out by what the method sets out to do. We simply cannot distinguish between the cases described in (1a) above, so we may have distinct neural sources, but we may not. SSD does appear to summarize the possible sources into a small number.
   c. As such, I think Figure 1 should suggest this more clearly that the sources extracted under SSD are representative, or perhaps equivalent, rather than genuine sources (see Nunez, Nunez, and Srinivasan, 2019, Brain Topogaphy).
   d. Line 78-79 also falls prey to this given that SSD can’t distinguish between the case of multiple coherent independent sources and the same source spreading to multiple electrodes so really the presence of non-zero weights across multiple electrodes only reveals a consistent rhythm measured across all of them without it necessarily being the same rhythm.
3. There are multiple times that source reconstruction is referenced however, this is confusing as it sets up two different goal posts that are alternatively targeted (data-driven referencing vs. agnostically identifying a putative set of representative neural sources). Further, if the goal is to have an approach that approximates a small number of sources from the activity at the electrodes, then multiple alternative approaches should also be demonstrated and compared here akin to Cohen (2017). Additionally, given that source reconstruction involves determining location information while incorporating tissue conductivity details, the suggestion that this method identifies sources akin to source reconstruction (as suggested in Figure 1) is a misnomer. I think adding in the caveat that they are representative sources potentially might help with this as well.
4. In several places I note that there is a desire to draw conclusions about both the data and the benefits of the algorithm at the same time. This, to me, is a difficult inference to perform since ground truth in data is unknown.
   a. In Section 3.1 the conclusion is drawn that since different components from SSD have different spatial patterns (different weights at the same electrode) and slightly different spectra (primarily differing in the 1/f), there are different rhythms at the same electrode. Does past simulation work back this up? Can SSD components be interpreted in this way? I am unclear why this is necessarily true, and why it could be the same rhythm with slightly different noise profiles say. Perhaps a deeper discussion of the implications of the generalized eigenvalue decomposition and what components mean under it (in Section 2.2.2) would clarify this immediately.
   b. Lines 296-297 suggests that SSD better uses information from the electrodes than other reference strategies. Yes, this is true as SSD is data-driven, but how do the results from the data provide evidence towards this?
   c. In line 312, the conclusion that SSD ‘enables recovering of rhythms in a flexible way regardless of location and dipole orientation’ seems an overextended interpretation of the results, this seems to be a conclusion we could have only in the presence of a ground truth (say in a simulation) or a very strong prior. Clearly SSD is agnostic to the biophysical details involved but I’m unsure whether the data can bear this out.
   d. Lines 338-340, this is perhaps something that would be best justified through a simulation/this is a line that might be better in the Discussion with an additional line pointing out that research to define the boundaries of what is possible to be discovered with this method in iEEG through simulations is critical for interpretation of its application.
5. For Section 3.4, would adding a figure showing the SNR distribution (w/t thresholding) for some components within predefined frequency bands (delta/theta/lower beta) be useful to further emphasize the point that a small set of subjects can drive overall results? If the SNR distribution has a long tail, this would be evidence in favor of this hypothesis.

**Smaller scale issues:**

1. Line 37, ‘used here are’ -> ‘used are’
2. In lines 195/196, it is quite plausible for the covariance matrix to be not full rank here, so is there some regularization applied? In the code I see that it says it expands the matrix in this situation, expressing that in the Methods in an equation/a line seems important.
3. In line 217, what exactly is being inverted here, C*W?
4. In lines 220 to 222, it’s unclear why a subset of electrodes based on a distance metric from the maximum location are chosen.
5. Line 255, what is the band range used to define the noise?
6. Line 295: ‘which utility’ -> ‘whose utility’
7. Lines 310-311: ‘Using SSD results in less bias’ -> Bias in a statistical sense refers to a difference from the expected mean, so I was unsure how it was being used here. I do agree that SSD makes fewer assumptions about the nature of how sources are distributed/how they are getting mixed and so this terminology might be better fit or perhaps just a definition of what is intended in using the word ‘bias’.
8. While a paragraph in the results is dedicated to discussing the possibly ways we could apply statistical tests to SSD in order to perform inference on the components (lines 343 - 347), why isn’t it implemented here? I suppose SSD is being demonstrated here as a data exploration tool, in that case this being the reason no statistical tests are implemented needs to be made explicit in this section or earlier in the Methods.
9. Line 353: what threshold of SNR was applied? 5 dB?
10. Lines 370-371: I understand this is of concern because of the possible effects of a few outliers on results, but that needs to be made explicit in this line.
11. In Figure 5, I was unclear what is meant when there’s no sphere in 5A, no dominant frequency present at that electrode?
12. In Figure 5B how is it possible for multiple components to have peak frequencies so close to one another? i.e. don’t the bandpass filters overlap considerably for some of these components?
13. Line 404: ‘am’ -> a
14. Line 450, same question about bias as in Q7, also I think there is one other time bias is used this way.

I hope the comments are helpful and want to reiterate that I think this is valid and useful contribution.
